# Differences in Neuropathic Pain and Radiological Features Between AQP4-ON, MOG-ON, and IDON

**DOI:** 10.3389/fpain.2022.870211

**Published:** 2022-05-09

**Authors:** Hao Kang, Huaiyu Qiu, Xiaofeng Hu, Shihui Wei, Yong Tao

**Affiliations:** ^1^Department of Ophthalmology, Beijing Chaoyang Hospital, Capital Medical University, Beijing, China; ^2^Senior Department of Ophthalmology, The Third Medical Center of PLA General Hospital, Beijing, China

**Keywords:** neuropathic pain, optic neuritic (ON), AQP4-ON, MOG-ON, IDON

## Abstract

**Purpose:**

The purpose of this study was to investigate pain and radiological features of different types of first-episode demyelinating optic neuritis (ON).

**Methods:**

Eighty-three patients presenting with first-episode aquaporin-4 (AQP4) antibody-associated ON (AQP4-ON; *n* = 28), myelin oligodendrocyte glycoprotein (MOG) antibody-associated ON (MOG-ON; *n* = 26) and idiopathic demyelinating optic neuritis (IDON, *n* = 29) were included in this retrospective case-control study. We assessed optic nerve lesions on magnetic resonance imaging (MRI), acute pain associated with onset of optic neuritis and clinical characteristics of those ON patients with different serum autoantibody status.

**Results:**

24 AQP4-ON patients (85.75%), 23 MOG-ON patients (88.5%) and 24 IDON patients (82.8%) suffered from ON-associated pain. MOG-ON had mostly retro-orbital pain; AQP4-ON and IDON had mostly neuropathic pain. In addition, pain was more severe in AQP4ON patients than in other ON patients. In MRI, bilateral involvement was more common in AQP4-ON than IDON (26.9 and 3.7%); radiological optic nerve head swelling was more common in MOG-ON than in AQP4-ON and IDON (68.0 vs. 23.1 vs. 25.9%). MRI lesion in peri-optic nerve sheath was more common in AQP4-ON (53.8 vs. 16.0 vs. 3.7%). In 70 patients with ON-associated pain, gadolinium enhancement of orbital optic nerve was most common in MOG-ON patients (82.4 vs. 55.0 vs. 33.3%, *P* = 0.018), and enhancement of optic chiasma was most common in AQP4-ON patients (40.0 vs. 5.9 vs. 6.7%, *P* = 0.015). Perineural and orbital enhancement was observed only in patients with MOG-ON (*P* < 0.001). The length of enhancement was longer in AQP4-ON patients than in MOG-ON and IDON patients.

**Conclusion:**

Pain is a common symptom in patients with all types of demyelinating ON. AQP4-ON is frequently associated with severe ON-associated pain and longitudinally extensive optic nerve inflammatory lesions. Intra-orbital and peri-optic inflammation were more frequently observed in patients with MOG-ON, which was closely related to optic disc swelling and retro-orbital pain provoked by eye movements.

## Introduction

Optic neuritis (ON) is an acute, inflammatory process that causes demyelination of the optic nerve and often leads to varying degrees of permanent visual impairment. ON can be an isolated event or the initial symptom of central nervous system (CNS) autoimmune-mediated demyelinating diseases, such as multiple sclerosis (MS) or neuromyelitis optica (NMO) or myelin oligodendrocyte glycoprotein antibody disease (MOG-AD).

Due to the varied pathogenesis, clinical characteristics, therapy and prognosis, NMO-ON, MOG-ON and MS/IDON (idiopathic demyelinating optic neuritis) are increasingly becoming recognized as distinct disease entities. For example, the glial autoantibodies associated demyelinating ON, anti-aquaporin 4 (AQP-4) in NMO and anti-MOG, are associated with unique clinical and radiologic features and varying responses to treatment compared with MS or IDON.

Pain in inflammatory and demyelinating CNS diseases can be prevalent and disabling, lead to a lower quality of life, and result in an increased healthcare burden. As in ON patients, pain is a common and disabling symptom and is often refractory to treatment (1). Patients with ON always present with more than one pain syndrome (i.e., one retro-orbital pain and one neuropathic pain) with complicated pathophysiological mechanisms. Because of different pathologic entities, the acute pain syndrome would be also different between ON patients. Despite the recent research advances in ON treatment, acute pain management in ON has proved challenging and marked by insufficient attention and low analgesic effect therapy.

In this study, we systematically evaluated acute pain of patients suffering from AQP4-IgG seropositive ON, MOG-IgG seropositive ON, and IDON, with special attention to the comparison of pain syndromes and MRI characteristics in those patients.

## Materials and Methods

### Subjects

In this study, we retrospectively assessed 83 patients with unilateral or bilateral acute ON, all of whom were recruited from the Ophthalmology Department of the Beijing Chaoyang Hospital of the Capital Medical University and the Chinese People's Liberation Army General Hospital (PLAGH) from June 2017 and August 2020. ON was the first symptom in all the patients who fulfilled the diagnosis criteria of ON according to the optic neuritis treatment trial (ONTT) (1). The exclusion criteria were as follows: (1) the presence of compressive, vascular, toxic, metabolic, infiltrative or hereditary optic neuropathy; (2) the presence of hepatitis viral infection, human immunodeficiency virus (HIV) infection, syphilis, lymphoma, graft-versus-host disease, human T-lymphotropic virus Type I, or previous head or neck radiation. Those who had retinal lesions or other causative ocular diseases were excluded, too.

The 83 acute demyelinating ON patients included 28 AQP4-IgG seropositive patients, 26 MOG-IgG seropositive patients, and 29 IDON patients. All the patients underwent a detailed ophthalmic examination by professional ophthalmologists, including best-corrected visual acuity (BCVA), intraocular pressure, pupillary reactions in unilateral or bilateral asymmetric conditions, slit lamp examination, ocular fundus examinations, Humphrey visual field (VF) assessment, and optic nerve function tests. BCVA was tested by using a Snellen chart and was transformed into logarithm of the minimum angel of resolution (logMAR) values by using Petzold et al. (2) VA conversion method. If a VA was below 0.01, finger-counting (FC), hand motion (HM), perception of light (LP) and no perception of light (NLP) were tested, and the results were documented accordingly.

Serum samples were collected during the acute phase of ON and within a month of recurrence and exacerbation. Patients were included only if the MRI confirmed lesion within the optic nerve within 30 days of symptom onset.

### AQP4-IgG and MOG-IgG Testing

All the serum samples were analyzed for the presence of AQP4-IgG and MOG-IgG by an extracellular live cell-staining immunofluorescence technique using cell-based assay (CBA) in human NMO/ MOG-transfected HEK293 cells. The samples were scored as positive or negative by at least 2 independent experiments. A dilution of 1:1,000 was employed as the maximum positive value and 1:10 as the cut-off for positive and negative cases.

### Pain Assessment

The concerning pain attributable to acute ON was recorded as described by patients according to frequency, intensity, location, characteristics and duration of the pain. The severity of pain from the patients was scored on a scale of 0 (no pain) to 10 (the severest pain subjects can imagine) according to a numerical rating scale (NRS). The patients were instructed not to describe or include any pain syndromes not directly due to onset of ON.

After reviewed by a neuro-ophthalmologist and a neurologist, pain symptoms associated with ON were recorded based on categorizations of retro-orbital pain, trigeminal neuralgia, headache, migraine, and mixed pain (having more than two of the four types of pain). The information of each patient's medical visits and prescription medications for pain was also reviewed. We have designated as retro-orbital pain any pain or discomfort localized to the retro-orbital or periorbital regions. Pain is classified as trigeminal neuralgia if sensory signs corresponding to the affected nervous structures are detected on examination. It always demonstrates a paroxysmal nature, often associated with very short-lived, electric-like, severe, stimulus-evoked pain (3). Headache and migraine were diagnosed according to The International Classification of Headache Disorders (4–6). Headache caused by ON is defined as pain behind one or both eyes caused by demyelination of the optic nerves and accompanied by impairment of central vision. Migraine is defined as severe throbbing pain or a pulsing sensation on one side of the head, which is often accompanied by nausea, vomiting, and extreme sensitivity to light and sound (7).

### Radiological Characterization

MRI scanning was performed on a 3-T MR scanner (Trio; GE Healthcare Europe GmbH, Freiburg, Germany). All the patients had standardized imaging, including axial T2 weighted and/or fluid attenuated inversion recovery (FLAIR) sequences, coronal T2 or FLAIR sequences of the whole brain, and coronal sequences with or without axial T2 fat suppressed (FS) sequences. T1-weighted axial and coronal sequences were performed following gadolinium administration and used to determine the location of abnormal enhancement. The axial views were used to determine the length of abnormal enhancement. The location and length of abnormal optic nerve enhancement on gadolinium-enhanced and fat-suppressed MRI were documented. Neuroradiology assessment was carried out by a neuroradiologist blinded to the patient's histories and diagnoses. To study the location and extent of lesion involvement, the optic nerves were divided into five subsets: orbital, canalicular, intracranial, chiasmal, and optic tract.

### Ethics Statement

This study was approved by the Ethics Committee of the Beijing Chaoyang Hospital and the PLAGH, and was conducted following the Declaration of Helsinki in its currently applicable version. Written informed consents were obtained from all the patients.

### Statistical Analysis

Statistical analysis was performed by using SPSS for Windows, Version 24.0. Continuous variables were analyzed using a non-parametric test (Mann-Whitney U test). The Chi-squared test, or Fisher's exact test if applicable, was used to analyze the categorical data. The differences among any three groups were identified by using the ANOVA or Kruskal-Wallis test. In order to reduce Type I errors, Bonferroni correction was applied to the *P*-values. The Spearman rank correlation coefficient was employed to statistically analyze the correlations between the pain severity and the number of Gd enhanced segments of lesions in MRI in all the patients with acute pain symptoms and Gd enhancement.

## Results

### Demographic Data and Clinical Characteristics

The demographic data and clinical characteristics of the 28 AQP4-ON, 26 MOG-ON and 29 IDON patients were compared (Table 1). The mean age at disease first onset was similar in the three groups. Female predominance was apparent in the AQP4-ON patients (89.3%) and the IDON patients (82.8%), but the MOG-ON patients were not characterized by more female patients, with the male to female ratio at 13:13. Pain is one of the most common symptoms in all the ON patients. All the three groups had a high proportion of ON-related pain, with no significant inter-group differences. The proportion of disc swelling and bilateral involvement did not differ between the three groups. Recurrent ON appeared frequently among the AQP4-IgG seropositive ON (27/28, 96.4%) and MOG-IgG seropositive ON patients (26/26, 100.0%), and the IDON patients (18/29, 62.1%) had a more frequent monophasic course (*P* < 0.001; AQP4-ON&MOG-ON: *P* > 0.05/3, AQP4-ON&IDON: *P* < 0.05/3; MOG-ON&IDON: *P* < 0.05/3). Seven patients (7/28, 25.0%) in the AQP4-ON group had a clinical history of myelitis, with myelitis occurring less frequently in the MOG-ON (1/26, 3.8%) and IDON (0/29, 0.0%) patients (*P* = 0.002). BCVA at attacks in the acute phase and BCVA recovery were compared. No differences in visual loss during the acute stage were observed between the three groups. At the last follow-up, the AQP4- ON patients were significantly more likely to get poor VA recovery over time than the other patients (*P* = 0.001).

**Table 1:** Epidemiologic and disease characteristics of ON patients.

	**AQP4-ON**	**MOG-ON**	**IDON**	***P*-value**
Number of patients	28	26	29	-
Age at onset (years)	36.86 ± 12.20	35.00 ± 12.93	33.34 ± 12.26	0.570
Gender (male: female)	3:25	13:13	5:24	0.003^**^
Ocular pain (*n*, %)	24/28 (85.7%)	23/26 (88.5%)	24/29 (82.8%)	0.925
Disc swelling (*n*, %)	11/28 (39.3%)	15/26 (57.7%)	11/29 (37.9%)	0.266
Bilateral, ever (*n*, %)	10/28 (35.7%)	9/26 (34.6%)	5/29 (17.2%)	0.238
Recurrent ON onset (*n*, %)	27/28 (96.4%)	26/26 (100.0%)	11/29 (37.9%)	<0.001^***^
Myelitis, ever (*n*, %)	7/28 (25.0%)	1/26 (3.8%)	0/29 (0.0%)	0.002^**^
BCVA at first ON attacks in acute time (logMAR)	2.16 ± 0.76	1.81 ± 0.78	1.78 ± 0.85	0.162
BCVA recovery at last follow-up (logMAR)	0.98 ± 0.72	0.36 ± 0.47	0.52 ± 0.59	0.001^**^

*ON, Optic neuritis; AQP4, Aquaporin-4; MOG, Myelin oligodendrocyte glycoprotein; IDON, Idiopathic demyelinated optic neuritis; MRI, Magnetic resonance imaging; BCVA, Best-corrected visual acuity.*

** *P < 0.01;*

*** *P < 0.001. The bold values are used to indicate values with P value < 0.05*.

### Comparison of Pain Characteristics Between AQP4-ON, MOG-ON and IDON

Type, severity, localization and treatment of ON-related pain were compared between different groups of ON (Table 2). ON-related pain was observed in more than 80% patients during the evaluation, and many patients had more than one pain syndrome. The three groups reported similar proportion in different pain types, suggesting no major difference in pain pattern. Retro-orbital pain, observed in more than 70% patients, was the most prevalent main pain syndrome in all the three groups. Pain provoked by eye movements was the most common type in those who had retro-orbital pain as their primary syndrome. The most common pain severity was moderate in MOG-ON and IDON patients. Severe pain, defined by a score of 7 or more of 10, was present in approximately half of the subjects with AQP4-ON (46.4%), but in the minority of the subjects with MOG-ON (11.5%) and IDON (13.8%) (*P* = 0.003). In the treatment of pain, the use of pain medication was more prevalent in the AQP4-ON patients than in the MOG-ON and IDON patients. However, only 21.4% of the AQP4-ON patients had a pain treatment, whereas the majority of the patients with pain did not have any pain medication.

**Table 2:** Pain characteristics in ON patients.

	**AQP4-ON**	**MOG-ON**	**IDON**	***P*-value**	**P1^**¶**^**	**P2^**¶**^**	**P3^**¶**^**
Pain	24/28 (85.7%)	23/26 (88.5%)	24/29 (82.8%)	0.925^‡^	-	-	-
Retro-orbital Pain	19/24 (79.2%)	21/23 (91.3%)	17/24 (70.8%)	0.223^‡^	-	-	-
Spontaneous	3/24 (12.5%)	4/23 (17.4%)	6/24 (25.0%)	0.642^‡^	-	-	-
Provoked by eye movements	13/24 (54.2%)	17/23 (73.9%)	16/24 (66.7%)	0.370^§^	-	-	-
Spontaneous and worsened by eye movements	11/24 (45.8%)	17/23 (73.9%)	14/24 (58.3%)	0.165^§^	-	-	-
Trigeminal neuralgia	16/24 (66.7%)	9/23 (39.1%)	15/24 (62.5%)	0.140^§^	-	-	-
Headache	8/24 (33.3%)	7/23 (30.4%)	3/24 (12.5%)	0.199^§^	-	-	-
Migraine	3/24 (12.5%)	5/23 (21.7%)	1/24 (4.2%)	0.173^‡^	-	-	-
Both types	1/24 (4.2%)	2/23 (8.7%)	0/24 (0.0%)	0.314^‡^	-	-	-
**Categorized pain severity**							
None (0)	4/28 (14.3%)	3/26 (11.5%)	5/29 (17.2%)	0.925^‡^	-	-	-
Mild (1-3)	4/28 (14.3%)	7/26 (26.9%)	6/29 (20.7%)	0.554^‡^	-	-	-
Moderate (4-6)	7/28 (25.0%)	13/26 (50.0%)	14/29 (48.3%)	0.119^‡^	-	-	-
Severe (7-10)	13/28 (46.4%)	3/26 (11.5%)	4/29 (13.8%)	**0.003** ^**§**^	<0.05/3	<0.05/3	-
Pain medications	6/28 (21.4%)	1/26 (3.8%)	2/29 (6.9%)	0.109^§^	-	-	-
**Other symptoms**							
Nausea and/or vomiting	8/28 (28.6%)	5/26 (19.2%)	2/29 (6.9%)	0.094^‡^	-	-	-
Photophobia	15/28 (53.6%)	13/26 (50.0%)	13/29 (44.8%)	0.823^§^	-	-	-

*§, Pearson's chi-squared test; ‡, Fisher's exact test. P1, AQP4-ON& MOG-ON; P2, AQP4-ON&IDON; P3, MOG-ON& IDON. ¶, Bonferroni method. The bold values are used to indicate values with P value < 0.05*.

### Radiological Differentiation of AQP4-ON, MOG-ON and IDON

**Figure 1:**
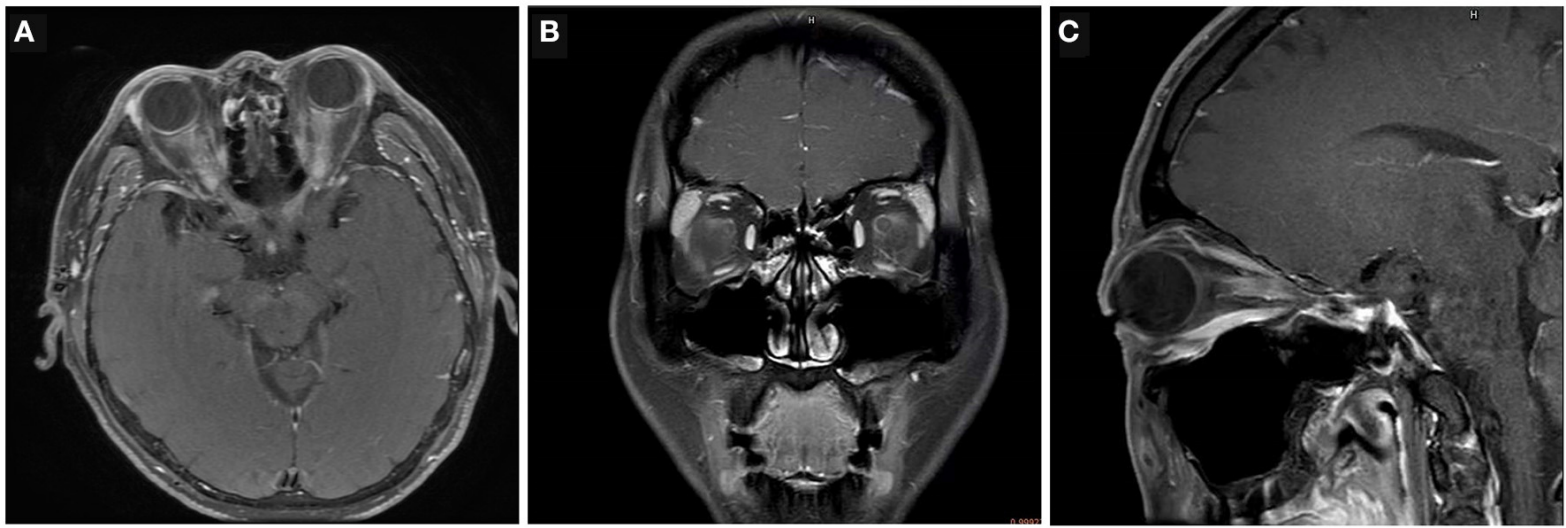
Examples of radiological characteristics in AQP4-ON, MOG-ON and IDON. **(A)** Bilateral longitudinally extensive optic nerve enhancement with optic chiasm involvement in a 43-year-old man with AQP4-ON. **(B)** Unilateral enhancement of the peri-optic nerve sheath in the left optic nerve in a 36-year-old man with acute MOG-ON. **(C)** Unilateral retrobulbar optic nerve enhancement and swelling in an 18-year-old woman with IDON.

More than 90% acute ON patients displayed abnormal optic nerves based on the MRI, including T2 lesions and gadolinium enhancing; however, there were no significant differences between the three groups (Figure 1). The presence of gadolinium enhancement also did not vary significantly between the three groups. Radiological bilateral ON (8, 9) was present in up to 20% of the patients with AQP4-ON and MOG-ON compared with a small minority of the patients with IDON (3.7%, *P* = 0.046). The radiological optic nerve head swelling (9, 10) in the MOG-ON patients appeared more prominent than in other types of ON patients. There was a significantly higher percentage of MOG patients (68.0%) with radiological optic nerve head swelling than paitents with AQP4-ON (23.1%, *P* < 0.05/3) and IDON (25.9%, *P* < 0.05/3) (Table 3).

**Table 3:** The radiological comparison of ON in patients with AQP4-ON, MOG-ON and IDON.

	**AQP4-ON**	**MOG-ON**	**IDON**	**P value**	**P1^**¶**^**	**P2^**¶**^**	**P3^**¶**^**
Lesions in MRI (*n*, %)	26/28 (92.9%)	25/26 (96.2%)	27/29 (93.1%)	>0.99^‡^	-	-	-
Bilateral lesion (*n*, %)	7/26 (26.9%)	5/25 (20.0%)	1/27 (3.7%)	**0.046** ^§*^	**-**	<0.05/3	**-**
Radiological optic nerve head swelling (*n*, %)	6/26 (23.1%)	17/25 (68.0%)	7/27 (25.9%)	**0.001** ^§**^	<0.05/3	**-**	<0.05/3
Lesion in peri-optic nerve sheath (*n*, %)	14/26 (53.8%)	4/25 (16.0%)	1/27 (3.7%)	**<0.001** ^§***^	<0.05/3	<0.05/3	**-**
Gadolinium enhancing (*n*, %)	21/26 (80.8%)	18/25 (72.0%)	17/27 (63.0%)	0.360^§^	-	-	-

ON, Optic neuritis; AQP4, Aquaporin-4; MOG, Myelin oligodendrocyte glycoprotein; IDON, Idiopathic demyelinated optic neuritis; MRI, Magnetic resonance imaging; BCVA, Best-corrected visual acuity. P1, AQP4-ON& MOG-ON; P2, AQP4-ON&IDON; P3, MOG-ON& IDON. §, Pearson's chi-squared test; ‡, Fisher's exact test; ¶, Bonferroni method. *P <0.05; **P <0.01; ***P <0.001. The bold values are used to indicate values with P value <0.05.

In this study, we also investigated the location and length of abnormal enhancement of the optic nerve in patients with different types of acute ON with pain (Table 4). In all the ON patients with acute painful symptoms, there were no significant differences in the proportion of abnormal gadolinium enhancement between the three groups. In the orbital segment, the MOG-ON (82.4%) patients had a higher proportion of involvement than the AQP4-ON (55.0%) and IDON (33.3%) patients (*P* = 0.018, MOG-ON & IDON: *P* < 0.05/3). In the MOG-ON patients, gadolinium enhancement predominantly involves the anterior segments of the optic nerve, with almost routine inclusion of the orbital segments, while chiasm and optic tract are generally spared. Chiasmal involvement was more common in the AQP4-ON patients than in the MOG-ON and IDON patients. Optic tract involvement was uncommon in all the groups, with AQP4-ON relatively more involved, accounting for 15.0%, but the MOG and IDON groups had no involvement. AQP4-ON tends to involve the posterior segments, including the optic chiasm and optic tract, more frequently. As to IDON, it tends to be more focal and limited in radiological involvement, with the number of affected segments in IDON significantly less than that in the AQP4-ON and MOG-ON patients (*P* = 0.003; AQP4-ON vs. IDON: *P* = 0.001, MOG-ON vs. IDON: *P* = 0.031). Inflammation and enhancement of the peri-optic nerve sheath, partly extending into the surrounding orbital fat, was only seen in MOG-ON patients. This appears crucial, as it was not apparent in either AQP4-ON or IDON patients, which may be one of the useful parameters for distinguishing MOG-ON from AQP4-ON and IDON. Further correlation analyses showed no significant correlation between the pain severity and the number of Gd enhanced segments (*P* = 0.764).

**Table 4:** Lesions in MRI of ON patients with pain.

	**AQP4-ON**	**MOG-ON**	**IDON**	**P value**	**P1^**¶**^**	**P2^**¶**^**	**P3^**¶**^**
	**(*n* = 24)**	**(*n* = 23)**	**(*n* = 24)**				
Gadolinium enhancing (*n*, %)	20/24 (83.3%)	17/23 (73.9%)	15/24 (62.5%)	0.262^§^	-	-	-
Orbital	11/20 (55.0%)	14/17 (82.4%)	5/15 (33.3%)	**0.018** ^§*^	-	-	<0.05/3
Canalicular	7/20 (35.0%)	8/17 (47.1%)	7/15 (46.7%)	0.775^§^	-	-	-
Intracranial	9/20 (45.0%)	3/17 (17.6 %)	3/15 (20.0%)	0.149^‡^	-	-	-
Chiasmal	8/20 (40.0%)	1/17 (5.9%)	1/15 (6.7%)	**0.015** ^‡*^	<0.05/3	-	-
Optic tract	3/20 (15.0%)	0/17 (0%)	0/15 (0%)	0.103^‡^	-	-	-
Perineural and orbital enhancement	0/21 (0%)	7/18 (38.9%)	0/17 (0%)	**<0.001** ^‡***^	<0.05/3	-	<0.05/3
Number of affected segments	1.90 ± 0.91	1.53 ± 0.51	1.07 ± 0.73	**0.003** ^†**^	0.226	0.001**	0.031*

ON, Optic neuritis; AQP4, Aquaporin-4; MOG, Myelin oligodendrocyte glycoprotein; IDON, Idiopathic demyelinated optic neuritis; P1, AQP4-ON& MOG-ON; P2, AQP4-ON&IDON; P3, MOG-ON& IDON; §, Pearson's chi-squared test; ‡, Fisher's exact test; ¶, Bonferroni method; †, Kruskal–Wallis H test. *P <0.05; **P <0.01; ***P <0.001. The bold values are used to indicate values with P value <0.05.

## Discussion

Pain is one of the most common comorbidities of ON, and it has varied degrees of impact on patient's quality of life (11). As documented in other ON cohorts (12–14), a large percentage (85.5%) of the ON patients in this study complained of ON-related pain. Despite the fact that all the patient groups described their pain experience differently, the pattern and prevalence of pain were similar. Retro-orbital pain affected most patients in all the groups, and most patients experienced more than one pain syndrome, which could alter over time even if there was no clinical relapse.

Demyelination lesions of the nervous system can cause eye pain, headache, extremities and trunk pain, and the mechanism underling are different (15–17): for example, the extremities and trunk pain are associated with the demyelination lesion in the spinal cord. Here in this study, demyelinating lesion mostly involved the optic nerves, which are not a part of nociceptive integrated pathway. The lesions on optic nerves induced wild spread inflammation, thus activating granulocyte infiltration, glia cells activity, and neuronal excitotoxicity, and resulting in neuropathic pain in these lesions. However, pain in ON patients caused by different etiologies may also have different pathophysiological mechanisms due to different target cells of demyelinating lesions.

In AQP4-ON, loss of AQP4, impairment of astrocytes and parallel injury in AQP4-coexpressed molecules (such as excitatory amino acid transporter 2, EAAT2), may lead to an excessive accumulation of glutamate in the extracellular space, interrupting the glutamine-glutamate-GABA axis (16). The balance between excitation and inhibition in nociceptive pathways, combined with impaired segmental and descending inhibition, may lead to neuropathic pain in AQP4-IgG seropositive patients. Recent studies have found that astrocytes can regulate nociceptive synaptic transmission via neuronal-glial and glial-glial cell interactions, and are involved in the modulation of pain signaling and the maintenance of neuropathic pain (18). The combination of these factors may be the cause of the most severe acute neuropathic pain in the AQP4-ON group (19). In this study, although the frequencies and types of pain did not differ significantly between the three groups, the severity of acute neuropathic pain—which could be predominantly influenced by the extent and degree of inflammation—was significantly higher in the AQP4-ON patients than in the other patients. The worst visual prognosis in the AQP4-ON group indicated that these patients had the most severe inflammatory injury. Combined with the orbital MRI results, the optic nerve was the most extensively affected in the AQP4-ON patients, which was thought to associated with visual field loss and visual involvement (20, 21). Previous studies also reported that the effectiveness of neuropathic pain medication was worse in NMOSD patients than in MS patients (22).

In MOG-IgG associated diseases, the loss of MOG and the damage of oligodendrocyte are caused by antibody-mediated destruction. This further leads to the accumulation of large amounts of nerve growth factor (NGF) in the CNS, which may lead to aberrant sprouting of unmyelinated nociceptive fibers and cause chronic neuropathic pain (23). In this study, the frequencies of pain caused by eye movement (73.9%), radiographic optic nerve head swelling (68.0%), and orbital gadolinium augmentation (82.4%) were all higher in the MOG-ON patients than in the other patients. The MOG-ON patients manifested obvious pain provoked by eye movement and optic nerve swelling as well as severe inflammation involving the orbital tissue around the optic nerve. This result confirmed previous findings that pain provoked by eye movement is related to retro-orbital lesions caused by the traction of the common tendinous ring (12, 24). In addition, it may be intensified through the traction of the extraocular muscles, which is closely attached to the optic nerve sheath at the orbital apex (11).

Moreover, in the demyelinating lesions of all types ON patients, microglia plays a significant role in synaptic remodeling, connectivity, and network function changes in neuropathic pain. It also plays active roles in brain regions important for the emotional and memory-related aspects of chronic pain (25). The activation of microglia and recruitment of immunoinflammatory cells leads to the production and release of cytokines, chemokines and growth factors, all of which might exert pain induction and amplification effect (16, 26–29). For example, MOG-IgG can trigger the tightly-controlled FcR and BTK-driven microglia proliferative response (30). Astrocyte damage and microglial activation, induced by AQP4-IgG in NMO, may influence the microglia-astrocyte signaling pathway in neuropathic pain (25). Meanwhile, the complement system was identified as a prominently modulated pathway in neuropathic and inflammatory pain states. An upregulation of complement proteins including C1q has been observed in neuropathic mice, and has been associated with the activation and proliferation of microglia (31). The involvement of the complement system also plays an important role in the occurrence of persistent inflammatory pain and pain hypersensitivity (31, 32).

Due to the innervation of sensory nerves around the eye, the pain around the affected eye and accompanying ON is believed to result from irritation of the meninges surrounding the optic nerve (11, 14). The fusion of origins of the superior and medial recti with the nerve sheath at the orbital apex would contribute to local traction, as it has been demonstrated on MRI, explaining pain worsening during eye movement (21). The trigeminal nerve provides sensory innervation of the eye and periocular region, and recurrent branches of the trigeminal nerve also supply the intracranial dura, venous sinuses, and cerebral vessels (11, 21). Therefore, neuralgia radiating to the trigeminal innervation area is also one of prevalent pain syndromes (39.1–66.7%). While ON patients typically suffer from retro-orbital pain often provoked by eye movements, headache can also arise in the orbital, frontal, and temporal regions if inflammation spreads from the optic nerve to the surrounding meningeal nerve sheath containing nociceptive fibers of trigeminal origin (21, 24).

In addition, the location and length characteristics of optic nerve lesion in orbital MRI may be other useful parameters for differentiating different subtypes of ON. MOG-ON was found to have a predominant anterior location, prominent optic nerve swelling, and severe intra-orbital perineural sheath leakage compared with other patients. Additionally, inflammation and enhancement of the perineural and orbital appeared exclusively in the MOG-ON cases, which were present in 38.9% of the MOG-ON patients, as it was not observed in the AQP4-ON or IDON patients. This orbital MRI characteristic can provide invaluable insights into the differential diagnosis of MOG-ON. By contrast, AQP4-ON often involves posterior segments such as the chiasm and optic tract but may also affect the intra-orbital and canalicular optic nerve. More importantly, the AQP4-ON patients tend to have significantly more extensive optic nerve lesions than other patients. Whereas, both the AQP4-ON and MOG-ON patients appear to have a higher incidence of bilateral ON, the IDON patients tends to be unilateral and more limited in radiological involvement.

Despite the fact that the types and severity of ON-related pain varied amongst the three groups, we found that the majority of the ON patients with pain are currently not effectively treated, which is consistent with the findings in previous research (17). Only <20% of the patients with pain in all the diagnostic categories received pain treatment. The AQP4-ON group had the highest proportion of patients receiving pain medication, but only 21.4%, and it had the highest proportion of severe pain. Fewer patients in the MOG-on and IDON groups received pain medication, 3.8 and 6.9%, respectively. Physicians and patients usually do not pay sufficient attention to painful symptoms of ON in the acute phase, which is one explanation for the low rate of pain medication usage. The patients who complain of severe pain seem more likely to receive pain medication. In addition, physicians may consider vision preservation and relapse prevention as key goals of treatment, ahead of treatment for symptoms such as pain. Many therapeutic intervention and treatment are often offered during the acute phase of ON, but little research has been done to confirm their effect on pain relief. Therefore, pain in ON deserves special attention during each office visit, and pain management should be a treatment goal in addition to disease control. Another reason for the low use of pain medications may be the presence of insufficient pain control despite treatment. NMOSD is often resistant to standard pain relief (16, 17, 33), leaving doctors with limited treatment options and believing that pain in NMOSD is refractory and inevitable. When a pain medication was not sufficient to control the symptoms, the possible side effects caused by the combination of pain medications are also a concern for doctors. This suggests that additional adjuvant strategies to control pain should be explored, because pharmacologic interventions may be insufficient. Thirdly, pain is difficult to quantify and self-reporting may be the best way to assess it, especially when disability evaluations focus on anatomical abnormalities and functional deficits rather than the impact of pain. There exists a large number of validated pain screening and assessment questionnaires, such as PainDETECT and SF-36 (34, 35), which are very helpful in screening patient's pain symptoms and assessing the impact of pain on function and emotion. However, most pain scales and questionnaires focus more on systemic pain symptoms, which are more likely to be caused by myelitis, and there are currently few standard methods for assessing and quantifying ON-related pain. These results show the necessity of exploring an effective, multimodal and multidisciplinary approach to pain assessment and management in ON patients.

Undeniably, this study also has its limitations. One drawback concerns the retrospective design and unintentional selection bias due to the recruitment of ON patients from referral centers. Another limitation is the lack of more detailed records of the different patterns and prevalence of pain syndromes in the patients with ON and its impact on their daily life. Thirdly, this study did not assess potential comorbidities that may have contributed to pain. In future studies, systematic recording, careful differentiation of different types of pain, and comprehensive evaluation of the efficacy of pain treatment are key points for effective observation and management in patients suffering from ON.

In conclusion, pain is a very frequent symptom in ON patients, often with a severe impact on the quality of life of AQP4-ON, MOG-ON and IDON patients. However, medication is currently insufficient in controlling pain, with only a small portion of ON patients receiving pain therapy and none of them being fully pain-free. In addition to relapse prevention, physicians should consider pain treatment as one of the therapeutic objectives in their future treatment of ON. Future prospective studies developing a further understanding in the multiplicity of pain syndromes and its pathophysiological mechanisms of ON-related pain may lead to better management and more rational therapy of pain in patients with ON.

## Data Availability Statement

The raw data supporting the conclusions of this article will be made available by the authors, without undue reservation.

## Ethics Statement

The studies involving human participants were reviewed and approved by the Ethics Committee of the Chinese People's Liberation Army General Hospital and the Beijing Chaoyang Hospital of the Capital Medical University. The patients/participants provided their written informed consent to participate in this study.

## Author Contributions

HK and YT: design and conduct of the study and preparation of the manuscript. HK, XH, and HQ: collection, management, analysis, and interpretation of the data. HK, SW, and YT: design and conduct of the study. Review and final approval of the manuscript by all authors.

## Funding

This work was supported by the National Natural Science Foundation of China (No. 81900849), the Beijing Hospital Authority Youth Programme (No. QMS20200308), and the National Key Research and Development Program (No. 2018YFE0113900).

## Conflict of Interest

The authors declare that the research was conducted in the absence of any commercial or financial relationships that could be construed as a potential conflict of interest. The reviewer LJ declared a shared affiliation with the authors HK, HQ, XH, and YT to the handling editor at the time of review.

## Publisher's Note

All claims expressed in this article are solely those of the authors and do not necessarily represent those of their affiliated organizations, or those of the publisher, the editors and the reviewers. Any product that may be evaluated in this article, or claim that may be made by its manufacturer, is not guaranteed or endorsed by the publisher.
